# Uptake, sequestration and tolerance of cadmium at cellular levels in the hyperaccumulator plant species *Sedum alfredii*

**DOI:** 10.1093/jxb/erx112

**Published:** 2017-04-12

**Authors:** Shengke Tian, Ruohan Xie, Haixin Wang, Yan Hu, Dandi Hou, Xingcheng Liao, Patrick H Brown, Hongxia Yang, Xianyong Lin, John M Labavitch, Lingli Lu

**Affiliations:** 1 MOE Key Laboratory of Environment Remediation and Ecological Health, College of Environmental & Resource Science, Zhejiang University, Hangzhou, China; 2 Department of Plant Sciences, University of California, Davis, CA, USA; 3 National Research Center for Geoanalysis, Beijing, China

**Keywords:** Cadmium, fluorescence microscopy, localization, micro X-ray fluorescence, protoplasts, tolerance, vacuole

## Abstract

*Sedum alfredii* is one of a few plant species known to hyperaccumulate cadmium (Cd). Uptake, localization, and tolerance of Cd at cellular levels in shoots were compared in hyperaccumulating (HE) and non-hyperaccumulating (NHE) ecotypes of *Sedum alfredii*. X-ray fluorescence images of Cd in stems and leaves showed only a slight Cd signal restricted within vascular bundles in the NHEs, while enhanced localization of Cd, with significant tissue- and age-dependent variations, was detected in HEs. In contrast to the vascular-enriched Cd in young stems, parenchyma cells in leaf mesophyll, stem pith and cortex tissues served as terminal storage sites for Cd sequestration in HEs. Kinetics of Cd transport into individual leaf protoplasts of the two ecotypes showed little difference in Cd accumulation. However, far more efficient storage of Cd in vacuoles was apparent in HEs. Subsequent analysis of cell viability and hydrogen peroxide levels suggested that HE protoplasts exhibited higher resistance to Cd than those of NHE protoplasts. These results suggest that efficient sequestration into vacuoles, as opposed to rapid transport into parenchyma cells, is a pivotal process in Cd accumulation and homeostasis in shoots of HE *S. alfredii*. This is in addition to its efficient root-to-shoot translocation of Cd.

## Teaser

Efficient vacuolar storage of Cd in parenchyma cells, as opposed to its rapid uptake, represents a pivotal process in Cd homeostasis and accumulation in shoots of the Cd hyperaccumulator *Sedum alfredii*.

## Introduction

Metal hyperaccumulation and the associated hypertolerance is a naturally selected, extreme and complex physiological trait found in a very small number of plant species (Verbruggen *et al.*, 2009; Krämer, 2010). The hyperaccumulators, which can accumulate extremely high levels of heavy metals in their leaves (Baker *et al.*, 2000, van der Ent *et al.*, 2013), are valuable for understanding fundamental aspects of metal homeostasis in plant cells (Clemens *et al.*, 2002; Pollard *et al.*, 2002; Clemens, 2006) and for their potential use in phytoextraction of metal-contaminated soils (McGrath and Zhao, 2003; Pilon-Smits, 2005; Zhao and McGrath, 2009). Cadmium (Cd) hyperaccumulation in plants is a very rare phenomenon due to its nonessential nature and its high toxicity. As a result only a few species have been identified as Cd hyperaccumulators (Krämer, 2010; Meyer and Verbruggen, 2012). Exploring the processes involved in Cd accumulation in the above ground parts of hyperaccumulators is helpful in revealing natural genetic variations in plant development, physiology, and adaptation under metal stress (Alonso-Blanco *et al.*, 2009; Krämer, 2010), as well as in optimizing plant-based strategies for soil remediation (Chaney *et al.*, 2007). To date, many studies on the mechanisms of heavy metal uptake, transport, accumulation and tolerance have been performed in hyperaccumulators, mainly in Brassicaceae plants (Verbruggen *et al.*, 2009; Krämer, 2010; Küpper and Leitenmaier, 2013). Efficient root uptake, enhanced root-to-shoot translocation, as well as the extraordinary storage capacities of shoot cells have been indicated as important factors for metal hyperaccumulation in plants. Currently, there is only limited information and a poor understanding of the processes of Cd absorption, accumulation and homeostasis at both cellular and subcellular levels in the shoots of hyperaccumulators.

*Sedum alfredii* Hance (Crassulaceae) is a zinc-cadmium (Zn/Cd) hyperaccumulator and a lead (Pb) accumulator, which is native to China (Yang *et al.*, 2002; Yang *et al.*, 2004; Tian *et al.*, 2010). It has significant potential for usage in the phytoremediation of polluted soils (Wu *et al.*, 2007; Wang *et al.*, 2012; Xiao *et al.*, 2013). Plants of the hyperaccumulating ecotype (HE) of *S. alfredii* grow naturally in a Pb/Zn mined region where the Cd concentration was up to 400 mg kg^-1^ in soil. Yang *et al.* (2004) reported that this ecotype of *S. alfredii* grew healthy in hydroponics at Cd levels up to 200 μM, whereas its non-hyperaccumulating ecotype (NHE) cannot survive at 50 μM Cd (Xiong *et al.*, 2004). It has been shown that HE *S. alfredii* exhibits an extraordinary capacity to translocate Cd from roots to shoots (Lu *et al.*, 2008) and can accumulate as high as 4,000 µg g^-1^ Cd in its aerial parts (Tian *et al.*, 2011). Such a high level of Cd translocation from roots to shoots in *S. alfredii* requires accessible, high capacity metal storage sites to sequester the excess metal ion for detoxification in plant cells. Studies thus far suggest that metal sequestration in most hyperaccumulators mainly occurs in non-photosynthetic cells of the epidermis and surface structures, namely trichomes, as a mechanism to reduce accumulation in the metal sensitive photosynthetic apparatus (Krämer, 2010; Küpper and Leitenmaier, 2013). In *S. alfredii* however, Tian *et al.* (2011) reported that Cd concentrations were highest in stem parenchyma and leaf mesophyll tissues, suggesting that compartmentalization of Cd in parenchyma cells may be important for the hyperaccumulation and detoxification of Cd. A better understanding of the mechanisms involved in the uptake and storage of Cd by the parenchyma cells is therefore necessary to elucidate the physiological mechanisms of Cd hyperaccumulation in *S. alfredii* and to provide insights into the diversity of strategies present in Cd hyperaccumulators.

The study of the uptake and sequestration of metals into plant cells is much more difficult to carry out than in animal and bacterial cells, which are devoid of cell walls (Leitenmaier and Küpper, 2011). Hence there is only limited information available on absorption and sequestration of heavy metals into specific storage cells of hyperaccumulators. The aim of the present study is to understand the characteristics of cellular uptake and sequestration of Cd into the terminal storage sites of the Cd hyperaccumulator *S. alfredii* (HE) in comparison with its non-hyperaccumulating ecotype (NHE). To reach this goal, we investigated: (1) the sequestration kinetics of Cd by plant cells in aerial parts of HE *S. alfredii* using micro X-ray fluorescence (µ-XRF), which is a powerful tool for spatial elemental imaging in biological systems (Pushie *et al.*, 2014); (2) Cd accumulation in individual leaf cells, namely protoplasts, isolated from the two contrasting ecotypes; (3) Cd tolerance in the two kinds of leaf cells in terms of cell viability and membrane integrity, as well as hydrogen peroxide (H_2_O_2_) production, under Cd stress.

## Materials and methods

### Plant culture

The plants of HE *S. alfredii* were obtained from an old Pb/Zn mine area in Quzhou, China. Those of NHE *S. alfredii* were obtained from a tea plantation in Hangzhou, China. Both locations are in the Zhejiang Province. Plants were grown in non-contaminated soil for more than three cycles of reproduction of shoot branches, in order to minimize the internal metal contents for HE *S. alfredii*. Uniform and healthy shoot branches were then cut and cultured hydroponically for root growth. After 2 weeks, rooted seedlings were subjected to 4 d exposures of one-fourth- and then one-half-strength nutrient solution. Plants were then cultured in full-strength nutrient solution containing 2.0 mM Ca^2+^, 4.0 mM NO_3_^-^, 1.6 mM K^+^, 0.1 mM H_2_PO_4_^-^, 0.5 mM Mg^2+^, 1.2 mM SO_4_^2-^, 0.1 mM Cl^-^, 10 μM H_3_BO_3_, 0.5 μM MnSO_4_, 5.0 μM ZnSO_4_, 0.2 μM CuSO_4_, 0.01 μM (NH_4_)_6_Mo_7_O_24_ and 100 μM Fe-EDTA. The nutrient solution pH was adjusted daily to 5.8 with 0.1 M NaOH or HCl. Plants were grown in a growth chamber with a 16 h: 8 h photoperiod at 400 μmol m^-2^ s^-1^, day: night temperatures of 26 ^o^C: 20 ^o^C, and day: night humidity of 70%: 85%. The nutrient solution was continuously aerated and renewed every 3 d.

### Elemental mapping of Cd by μ-XRF

#### Sample preparation

After pre-culturing for 4 weeks, seedlings of HE and NHE *S. alfredii* were treated with 100 μM CdCl_2_ in nutrient solutions. Plants were harvested following 7 d of exposure and fresh stems and leaves were cut and rinsed. The mid-transverse areas of stem and leaf samples at similar developmental stages were selected from both ecotypes for comparison. The mid-stem cross-sections were also collected from HE *S. alfredii* after exposure to 10 μM Cd for 7 d and 14 d. Using a cryotome (LEICA, CM1950), 100 µm thick sections of all the samples were cut. The plant samples were frozen with liquid nitrogen and fixed immediately on the specimen disks using a cryo-embedding OCT compound (Tissue Tek^®^) on the actively cooled (-40 ^o^C) speciation quick freezing shelf. The samples were then sectioned at a temperature of -20 ^o^C and freeze-dried at -20 °C for 3 d (Tian *et al.*, 2010; Tian *et al.*, 2011).

#### μ-XRF analysis

μ-XRF analysis of Cd, phosphorous (P), sulphur (S) and chlorine (Cl) within plant samples was carried out on a SSRL beamline 14-3, USA, as previously described (Tian *et al.*, 2016). The incident X-ray beam of 5 μm in the beamline 14-3 was focused using a pair of Kirkpatrick-Baez mirrors. The incident beam was monochromatized using a Si(111) double-crystal monochromator. For the collection of μ-XRF maps, the beam energy was set to 3.55 keV, which is above the Cd L_3_ edge and below the potassium edge, to avoid the strong potassium signal and so allow detection of the elements P, S, Cl and Cd. The samples were filled with helium. The fluorescence yield was detected using a single channel Vortex Si detector. μ-XRF maps were obtained by rastering the beam at 10 μm steps, with a count time of 100 ms per step. The spectra were calculated using the software package SMAK, version 0.34, S-4 (http://www.ssrl.slac.stanford.edu/~swebb/smak.htm).

To further confirm the spatial distribution of Cd in stems of HE *S. alfredii*, μ-XRF mapping was performed on stems at different developmental stages from plants treated with 100 μM Cd for 30 d. These stem samples were analysed on a beamline 13-ID-C (GSECARS) at the Advanced Photon Source, USA, as previously described (Tian *et al.*, 2011). The incident beam was set at 30 keV and the spot size of the X-ray beam was set up to 2*2 µm^2^ at 5 μm steps, with a count time of 0.1 s per step. The fluorescence energies windowed for this investigation were potassium (K), calcium (Ca), manganese (Mn), iron (Fe), Zn and Cd.

### Preparation and purification of mesophyll protoplasts

Plants of intact 4-week old seedlings of HE and NHE *S. alfredii* were selected for mesophyll protoplast isolation. Protoplasts were prepared based on the method developed by Ma *et al.* (2005) and Robert *et al.* (2007), with some modification. Briefly, uniform leaves were selected and peeled to remove abaxial sides. They were then sliced into 1 to 2 mm pieces and suspended in 30 ml cell digesting medium composed of 1.5% (w/v) cellulase, 0.4% (w/v) macerozyme, 0.4 M mannitol, 20 mM KCl, 20 mM MES and 10 mM CaCl_2_. After collection by vacuum filtration, the suspension was shaken at 60 rmp at 28 ^o^C for 2 h. Afterwards, the suspension was filtered through a 75 μm cell strainer and rinsed twice with W5 buffer, which comprised 154 mM NaCl, 125 mM CaCl_2_, 5 mM KCl and 2 mM MES at pH 5.8. The collected protoplasts were then centrifuged at 80 g for 20 min in a J2-HS centrifuge (Beckman Coulter). The pellets were suspended in 30 ml W5 buffer and centrifuged twice under the same conditions to eliminate the enzymes. Isolated mesophyll protoplasts were suspended in W5 buffer for use in the following experiments.

### Determination of protoplast integrity

Protoplast density was measured via a viability test using fluorescein diacetate (FDA) dye with a hemocytometer under fluorescence microscopy (Nikon-Eclipse-E600, Japan). The viable protoplasts were spheroid and showed bright green fluorescence, whereas dead protoplasts were ruptured. A 3 ml protoplast solution was suspended in W5 buffer, supplemented with or without Cd. At each time interval (0–7.5 h), a 10 μl suspension was collected for viability analysis. The percentage viability in the Cd exposed samples was calculated by normalization to the number of viable protoplasts from the control.

### Time-course kinetics of Cd accumulation in protoplasts

Mesophyll protoplasts isolated from both HE and NHE young leaves were incubated in W5 buffer. To protect the protoplasts from light, the samples were kept in the dark before the following experiments. An aliquot of a concentrated solution of CdCl_2_ was added to the W5 buffer to achieve a final Cd concentration of 10 μM. At each time interval (0–2 h), the solution was filtered through a 10 μm filter membrane, with the protoplasts retained on the membrane due to their diameter being larger than the aperture of filter membrane. The protoplasts were then gently rinsed three times with 4 ml W5 buffer to remove any Cd^2+^ on their surfaces. Each treatment was replicated four times. The protoplasts on the filter membranes were broken and dissolved using 15 mL deionized water and then filtered through a 0.45 μm membrane. The experiments were conducted at two different temperatures, 25 ^o^C and 4 °C. The concentrations of Cd in the filtrates were analysed using inductively coupled plasma mass spectroscopy (ICP-MS; Agilent 7500a, CA, USA). The integrity of mesophyll protoplasts was determined as described above at each time interval, in order to precisely calculate the Cd accumulation rate in the protoplasts.

### Concentration-dependent kinetics of Cd accumulation in protoplasts

Mesophyll protoplasts isolated from young leaves of the two *S. alfredii* ecotypes were compared in regards to the concentration-dependent kinetics of Cd accumulation. Collected protoplasts were incubated in W5 buffer in the dark and then supplemented with a series of concentrations of different CdCl_2_, ranging from 0 to15 μM, at two different temperatures, 25 °C and 4 °C. All the solutions were mixed carefully and used immediately. After 30 min for uptake, Cd concentrations in the protoplasts were analyzed as described above, with each treatment replicated four times. The Cd accumulation rate for each replicate was calculated using Cd concentration and protoplast integrity data.

### Microscopic imaging of Cd in protoplasts

The Cd-specific probe Leadmium^TM^ Green AM dye (Molecular Probes, Invitrogen, Carlsbad, CA, USA) was used to investigate Cd localization in protoplasts. Protoplasts were incubated in W5 buffer with the addition of 0.4 μg mL^−1^ Leadmium ^TM^ Green AM stock solution. The stock solution of Leadmium^TM^ Green AM was prepared by adding 50 μl of dimethyl sulphoxide (DMSO) to one vial of the dye and then diluting this with 1:10 of 0.85% NaCl (Lu *et al.*, 2008). After incubation for 30 min in the dark on a shaker set to 60 r min^-1^, the protoplasts were washed four times with fresh W5 buffer to remove extra Leadmium^TM^ Green AM dye. In each washing step, protoplasts were centrifuged at 50 g for 1 min and the supernatant replaced by an equal volume of fresh W5 buffer. After the last wash, the protoplasts were carefully mixed with W5 buffer, with addition of 10 µM or 200 µM Cd. At each time interval (0, 10, 30, 60, 90, and 120 min), the fluorescence of Cd in protoplasts was detected under a fluorescence microscope (Nikon-Eclipse-E600, Japan) using filters S450-490 for excitation and S505-520 for emission. For concentration-dependent imaging of Cd, the protoplasts were treated with different amounts of Cd (0, 5, 10, 20, and 30 µM) for 90 min and then imaged using fluorescence microscopy, as described above.

### Microscopic imaging of H_2_O_2_ in protoplasts

The detection of H_2_O_2_ in protoplasts *in vivo* was carried out using CM-H_2_DCFDA (Invitrogen, USA), according to the methods of Zhang *et al.* (2009), with slight modification. The protoplasts were suspended in W5 buffer with or without 200 μM Cd. At each time interval (10 min, 30 min, 1 h and 2 h), a 200 μl solution was collected and rinsed in W5 buffer three times to remove any Cd^2+^ on the protoplast surfaces. This solution was then incubated for 10 min in the dark with the addition of CM-H_2_DCFDA at a final concentration of 5 μM. As negative controls, protoplasts were incubated for 30 min with a H_2_O_2_ scavenger, namely 1.0 mM ascorbate (ASC), before staining with the fluorescent dyes (Tian *et al.*, 2012). H_2_O_2_ fluorescence was visualized using a 45 μl sample of the solution placed in single concave slide, using a fluorescence microscope (Nikon-Eclipse-E600, Japan), with excitation at 450-490 nm and emission at 505-520 nm. The relative fluorescence units were converted to percentages by normalization to the H_2_O_2_ fluorescence of the control.

### Statistical analysis of data

All data were statistically analysed using the SPSS package (Version 11.0). Analysis of variance (ANOVA) was performed on the data sets and the mean and SE of each treatment as well as LSD (*P* < 0.05 and *P* < 0.01) for each set of corresponding data were calculated.

## Results

### Cellular distribution patterns of Cd in stems and leaves

**Fig. 1.**
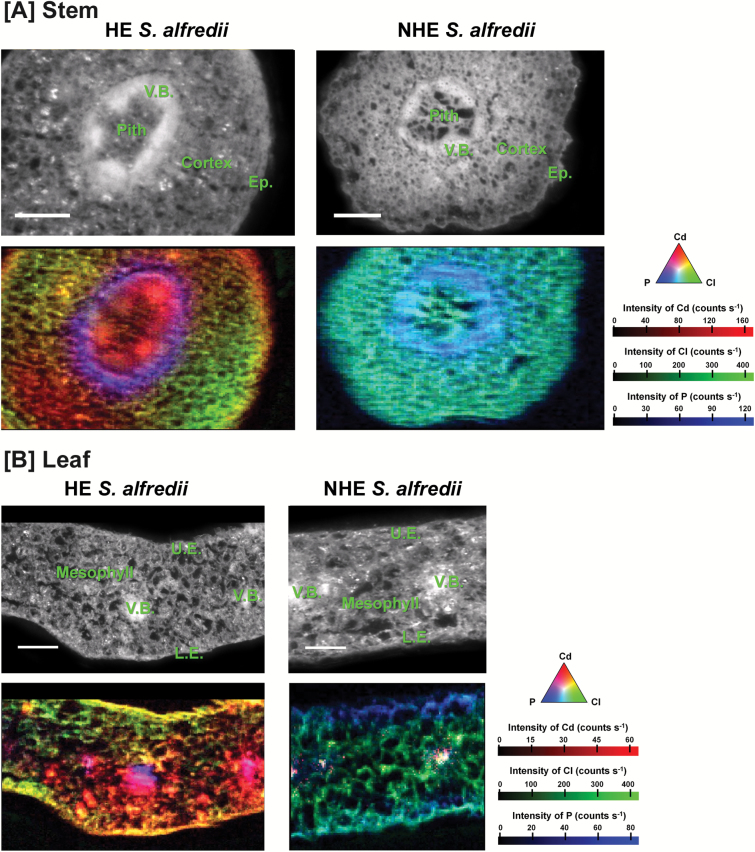
µ-XRF imaging of Cd (red), Cl (green) and P (blue) in stem (A) and leaf (B) cross-sections of HE and NHE *S. alfredii* treated with 100 µM Cd for 7 d. The brightfield images show the stem and leaf regions selected for μ-XRF images. Pixel brightness is displayed in RGB, with the brightest spots corresponding to the highest fluorescence (counts s^-1^) for the element depicted. Scale bar, 200 µm. V.B., vascular bundles; Ep., epidermis. L.E., lower epidermis; U.E., upper epidermis. (This figure is available in colour at *JXB* online.)

Plants of HE *S. alfredii* grew healthily after treatments of 10 or 100 µM Cd for 7–30 d, while NHEs did not grow after exposure to 100 µM Cd for 14 d (see Supplementary Table S1 at *JXB* online). The distribution patterns of Cd (red), Cl (green) and P (blue) are shown for the stem and leaf cross-sections from HE and NHE *S. alfredii* plants treated with 100 µM Cd for 7 d (Fig. 1). Pixel brightness for the elements is displayed in RGB, with the brightest spots corresponding to the highest fluorescence. The distribution patterns of Cd were very different between the two contrasting ecotypes of *S. alfredii*. The Cd signal intensity was up to 175 counts s^-1^ in the stem cells of HE, whereas very low Cd intensity was observed in the cross-sections of the stems from NHE plants treated with the same concentration of Cd (Fig. 1A). The μ-XRF images of Cd in leave samples collected from HE plants also showed enhanced localization of Cd to leaf vein and mesophyll tissues, when compared with the very low Cd signal restricted within vascular bundles in the NHE samples (Fig. 1B).

**Fig. 2.**
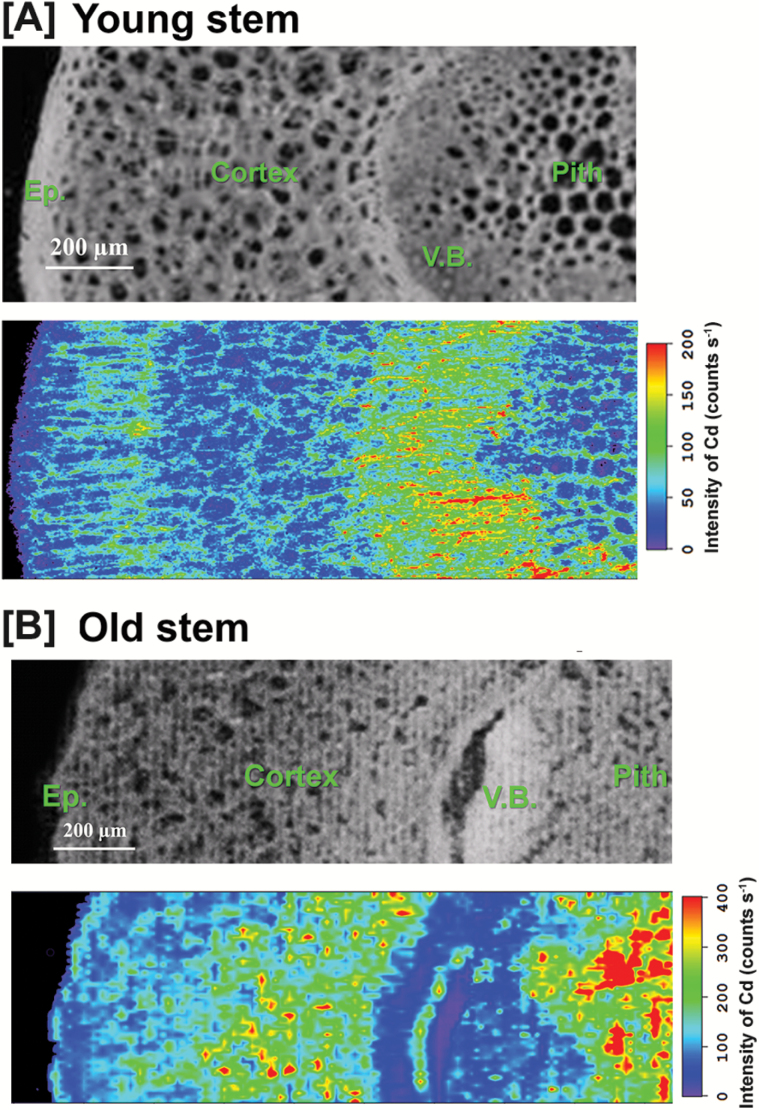
µ-XRF imaging of Cd distribution in young- tem (A) and old stem (B) cross-sections from HE *S. alfredii* treated with 100 µM Cd for 30 d. The brightfield images show the stem region selected for μ-XRF imaging. In each map red is scaled to the maximum value. Scale bar, 200 µm. V.B., vascular bundles; Ep., epidermis. (This figure is available in colour at *JXB* online.)

**Fig. 3.**
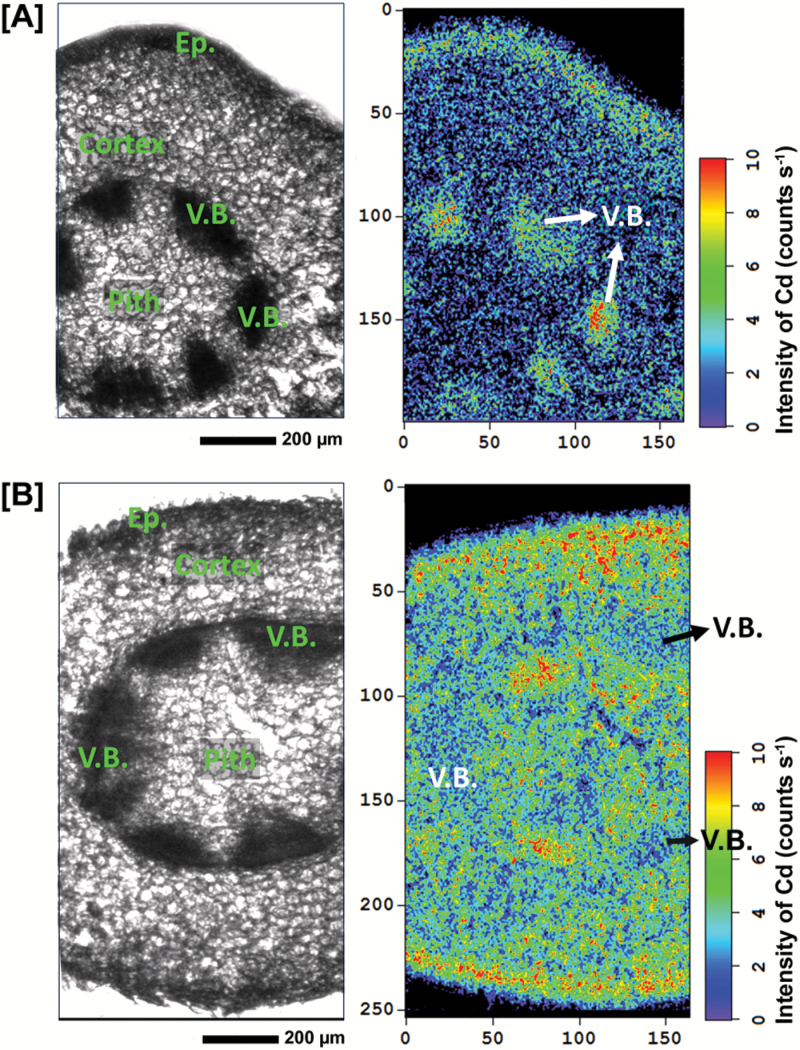
µ-XRF imaging of Cd distribution in the mid-stem cross-sections from HE *S. alfredii* treated with 10 µM Cd for 7 d (A) or 14 d (B). The brightfield images show the stem region selected for μ-XRF imaging. In each map red is scaled to the maximum value. Scale bar, 200 µm. V.B., vascular bundles; Ep., epidermis. (This figure is available in colour at *JXB* online.)

In the stem cross-sections of HE plants treated with 100 µM Cd for 7 d (Fig. 1A), Cd was preferentially distributed in the vascular tissues, but also highly localized to some cortex and pith tissues. Cd images for stems at different developmental stages from HE plants after 30 d Cd exposure showed an age-dependent difference in Cd distribution patterns (Fig. 2). In young stems, Cd was highly concentrated in the vascular bundles (Fig. 2A), while in old stems Cd preferentially localized to pith tissue and the cortex layer around the vascular bundles (Fig. 2B). The distribution patterns of Cd were also analyzed for the mid-stem cross-sections collected from HE *S. alfredii* plants treated with 10 µM Cd for 7 d and 14 d. The results showed the preferential localization of Cd in the stem vascular bundles after 7 d and the tendency for Cd movement into the epidermis, external parts of the cortex, and some areas in the pith as the exposure time increased to 14 d (Fig. 3).

### Time- and concentration- dependent kinetics of Cd accumulation in mesophyll protoplasts

**Fig. 4.**
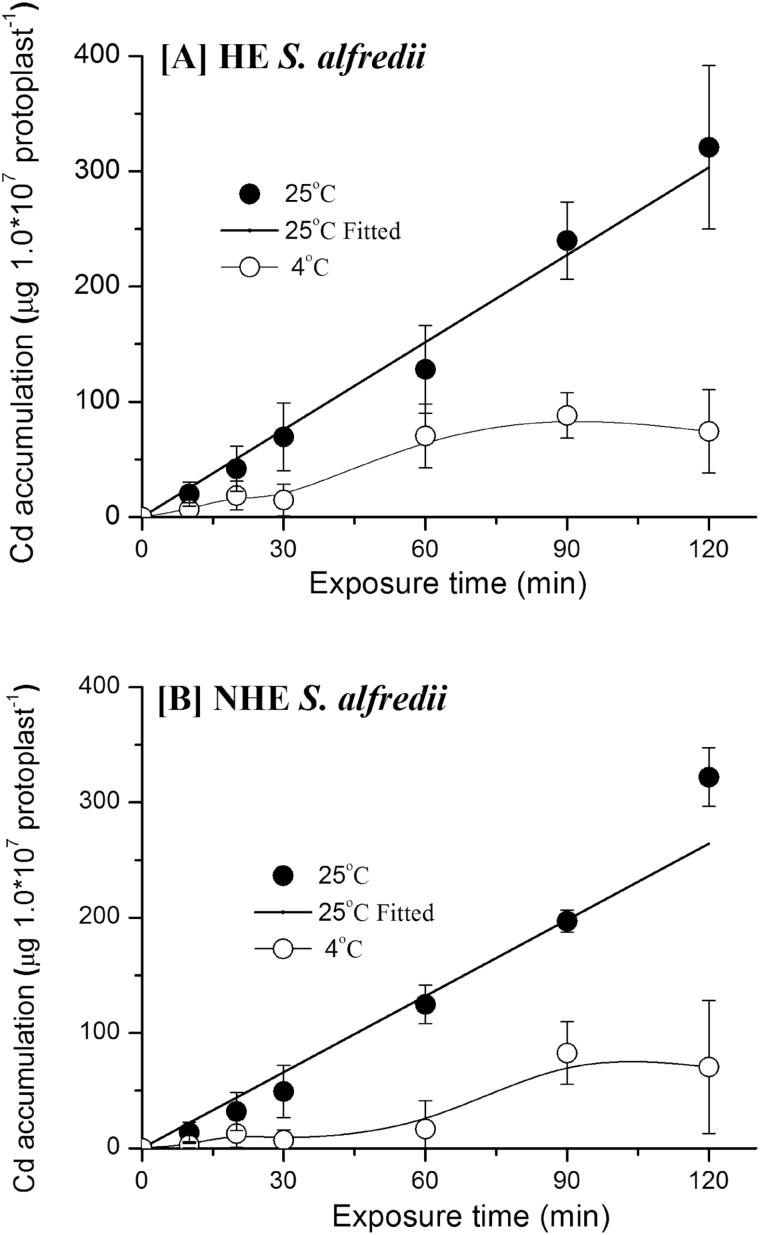
Time-dependent accumulation kinetics of Cd in mesophyll protoplasts of HE (A) and NHE (B) *S. alfredii*. The protoplasts were isolated from young leaves of 4-week old HE and NHE *S. alfredii* plants and then treated with 10 μM Cd at 4 ^o^C and 25 ^o^C for different time periods, as shown in the figure. Data points and error bars represent means (*n* = 5) and SE, respectively. Error bars do not extend outside some symbols.

Mesophyll protoplasts were successfully isolated from young leaves of HE and NHE *S. alfredii* plants (Supplementary Fig. S1). The protoplasts were exposed to uptake solutions containing 10 μM Cd for the given time-course for accumulation of the metal. The 2 h uptake period showed no significant difference in terms of the Cd accumulation rate between the protoplasts of the two ecotypes (Fig. 4). Cd accumulation in the mesophyll protoplasts of both ecotypes was more or less linear within the 2 h window of exposure to 10 μM Cd (Fig. 4). Ice-cold treatment at 4 °C significantly decreased the cumulative accumulation of Cd in both HE and NHE mesophyll protoplasts within the 2 h uptake period (Fig. 4).

**Fig. 5.**
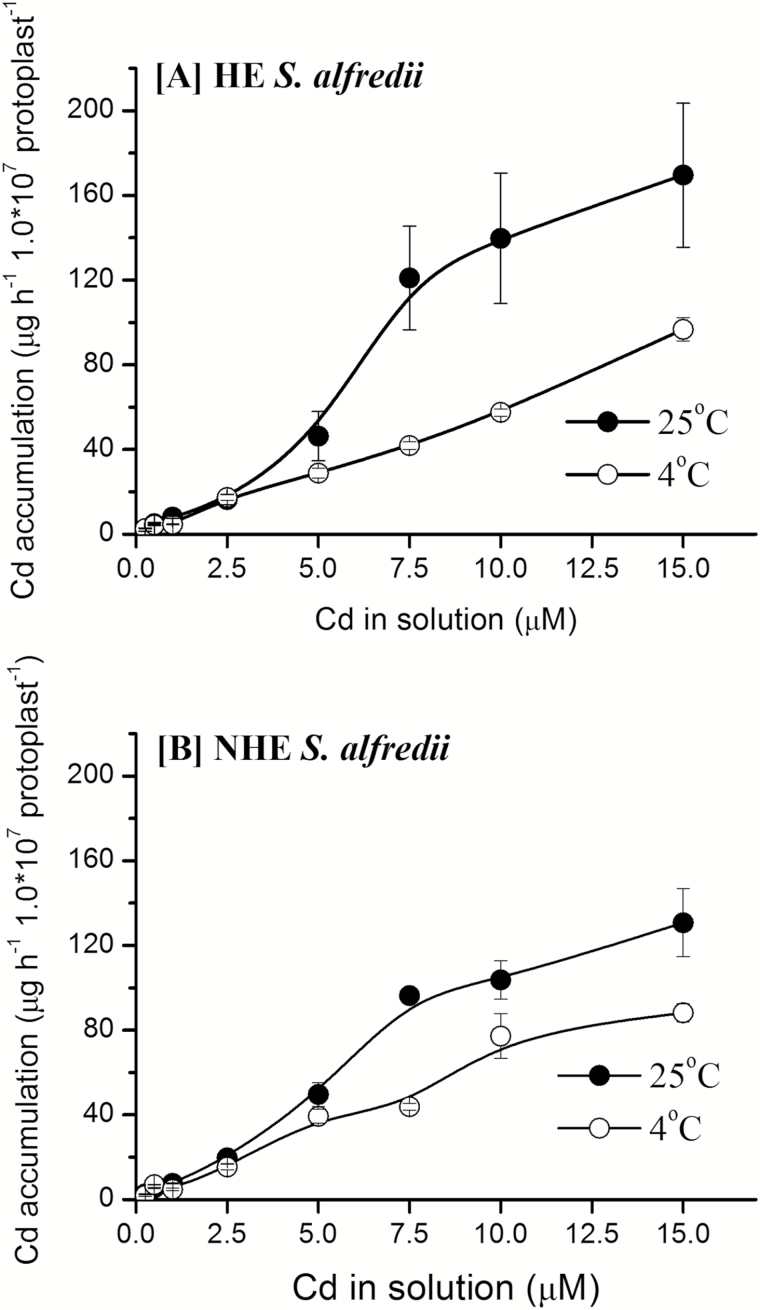
Concentration-dependent accumulation kinetics of Cd in mesophyll protoplasts of HE (A) and NHE (B) *S. alfredii*. The protoplasts were isolated from young leaves of 4-week old HE and NHE *S. alfredii* plants and then treated with different concentrations of Cd at 4 ^o^C and 25 ^o^C. Data points and error bars represent means (*n* = 5) and SE, respectively. Error bars do not extend outside some symbols.

Concentration-dependent Cd accumulation kinetics in mesophyll protoplasts were further investigated for the two *S. alfredii* ecotypes. At low Cd levels (0-15 μM) in solution, concentration-dependent Cd accumulation kinetics in mesophyll protoplasts from the both ecotypes were characterized by non-saturating curves, with the accumulation rate slightly but not significantly higher in HE protoplasts (Fig. 5). As with experiments into the time-dependent kinetics of Cd accumulation (Fig. 4), treatments at a low temperature of 4°C significantly inhibited Cd uptake by mesophyll protoplasts of both *S. alfredii* ecotypes, regardless of Cd levels in the uptake solutions. 

### Localization of Cd in mesophyll protoplasts

**Fig. 6.**
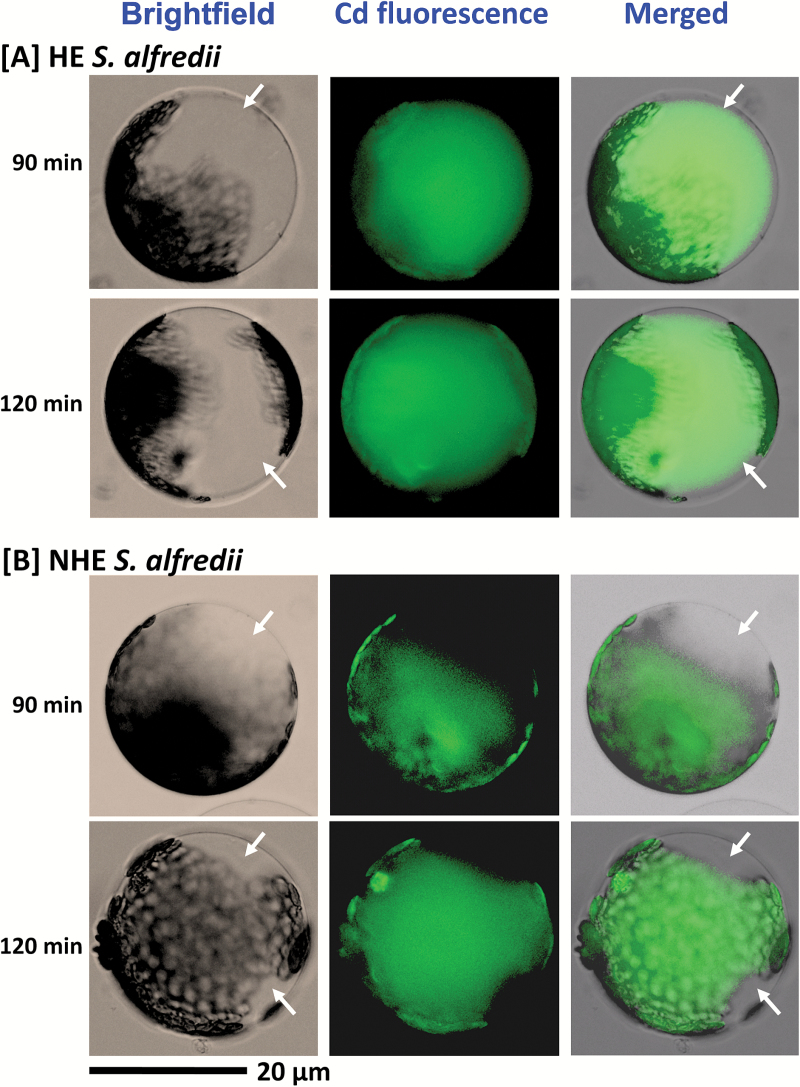
Imaging of Cd fluorescence in mesophyll protoplasts isolated from young leaves of HE (A) and NHE (B) *S. alfredii* using Leadmium^TM^ Green AM dye. The protoplasts were isolated from young leaves of 4-week old HE and NHE *S. alfredii*, pre-loaded with Leadmium^TM^ Green AM dye for 30 min, and treated with 20 µM Cd for 90 or 120 min. Green fluorescence represents the binding of the dye to Cd. Scale bar, 20 µm. (This figure is available in colour at *JXB* online.)

Fluorescence imaging of Cd in protoplasts revealed differences between the two *S. alfredii* ecotypes. The Cd probe, Leadmium^TM^ Green AM dye, was successfully loaded into the mesophyll protoplasts of both ecotypes treated with Cd, showing clear bright green fluorescence, with a very weak signal in controls (see Supplementary Fig. S2). After treatment with 10 µM Cd for 60–120 min, some mesophyll protoplasts collected from HEs were filled with Cd, as indicated by the clear bright green spheroid in the fluorescence images (see Supplementary Fig. S2A). Fluorescence imaging confirmed that the sequestration of Cd into vacuoles occurred in HE protoplasts treated with 10–30 µM Cd for 90 min (Supplementary Fig. S3). As shown in Fig. 6, protoplasts were spheroid with a large vacuole in the centre of the cells and chloroplasts distributed around their periphery. The merged images of Cd fluorescence plus brightfield indicated that Cd was largely localized centrally in HE protoplasts after a 90 min exposure to 10 µM or 20 µM Cd (Supplementary Fig. S4A, Fig. 6A). In contrast, green fluorescence of Cd was only present at the periphery of mesophyll protoplasts from NHEs. Furthermore a very low amount of Cd was taken up into the vacuoles of NHE protoplasts within the 120 min timeframe (Supplementary Fig. S4B, Fig. 6B). Treatment of mesophyll protoplasts with a high concentration of Cd (200 µM) confirmed the efficient sequestration of Cd by the vacuoles of HE plants (Supplementary Fig. S5). After a 10 min exposure to high levels of Cd, Cd fluoresence was observed in the centre of HE protoplasts, although its intensity was slightly lower than that in the other parts of the cell (Supplementary Fig. S5A). This suggests that a certain amount of Cd uptake is undertaken by mesophyll protoplasts of HEs, with Cd entering into their vacuoles within 10 min of exposure. The central localization of Cd in HE protoplasts became more clear and pronounced with longer exposure to Cd.

### Protoplast integrity under high Cd stress

**Fig. 7.**
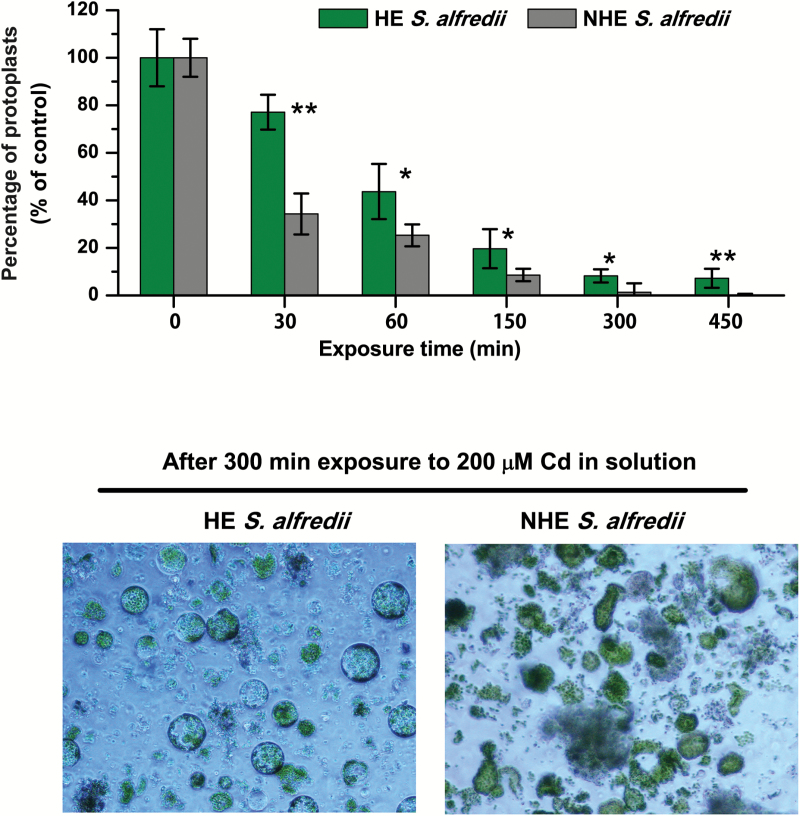
Integrity, as a percentage of control, of mesophyll protoplasts isolated from HE and NHE *S. alfredii* after exposure to 200 μM Cd for 0–450 min. The protoplasts were isolated from young leaves of 4-week old HE and NHE *S. alfredii* plants and treated with 200 μM Cd for different time periods, as shown in the figure. Data points and error bars represent means (*n* = 5) and SE, respectively. One and two asterisks indicate significant difference between HE and NHE at *P* < 0.05 and *P* < 0.01, respectively. (This figure is available in colour at *JXB* online.)

Protoplast integrity was monitored during their uptake of Cd through both ICP-MS and fluorescence imaging. Interestingly, the results showed that mesophyll protoplasts isolated from HEs appear to be more tolerant to Cd stress than those of NHEs. Protoplast integrity, as a percentage relative to controls, in HE *S. alfredii* was significantly higher than that of NHEs after exposure to 10 µM Cd for 2 h (Supplementary Fig. S6). This difference in tolerance between the HE and NHE protoplasts is highly pronounced under high Cd stress (Fig. 7). Regardless of the Cd treatment period, the percentage of intact protoplasts in HEs in the presence of Cd was consistently higher than that of NHEs. The estimated mean viability of NHE protoplasts was less than 40% after Cd exposure for only 30 min, with intact protoplasts scarcely observed at 5 h. Although the percentage of intact protoplasts of HE *S. alfredii* gradually decreased as Cd exposure time increased, about 10% of the protoplasts remained viable for at least 7.5 h (Fig. 7). According to the results of two-way ANOVA, the ecotypes, Cd exposures and their interactions had a significant effect (*P* < 0.001) on mesophyll protoplast integrity. Protoplast integrity and viability was also determined using FDA dye and showed a similar result, namely higher tolerance of HE protoplasts to high Cd stress when compared with NHEs (Supplementary Fig. S7).

### Intracellular ROS production

**Fig. 8.**
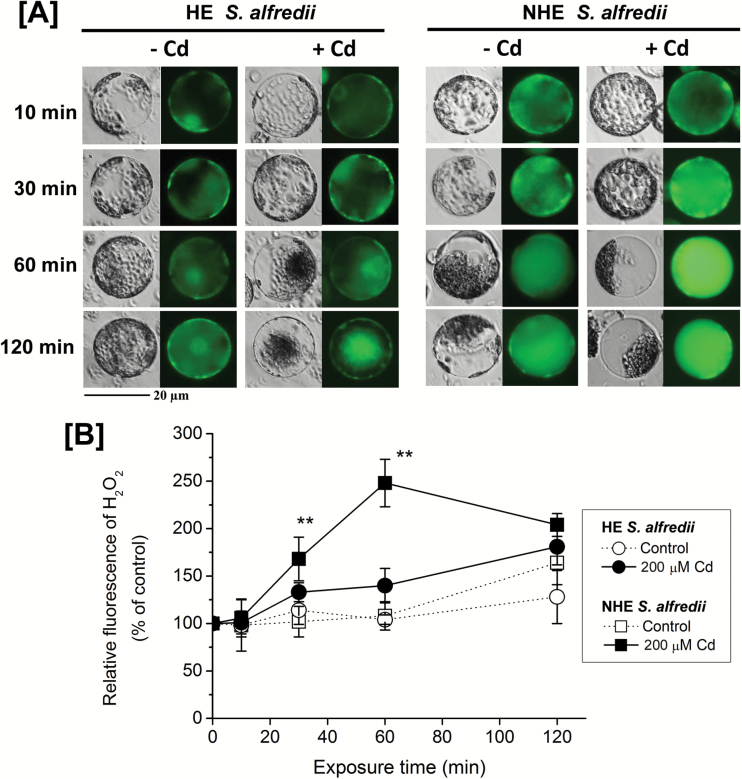
H_2_O_2_ levels in mesophyll protoplasts isolated from young leaves of HE and NHE *S. alfredii* in response to Cd stress. (A) Detection of H_2_O_2_ fluorescence using CM-H_2_DCFDA staining and ﬂuorescence microscopy. Scale bar, 20 μm. (B) H_2_O_2_ production expressed as relative ﬂuorescence. The protoplasts were isolated from young leaves of 4-week old HE and NHE *S. alfredii* plants, pre-treated with 200 µM Cd for 0, 10, 30, 60, and 120 min, and then loaded with 5 μM CM-H_2_DCFDA for 10 min. Representative images showing endogenous H_2_O_2_ concentrations are given. Data are means ± SD (*n* = 8–12). Two asterisks indicate significant difference between Cd and control treatments at *P* < 0.01. (This figure is available in colour at *JXB* online.)

Intracellular reactive oxygen species (ROS) production was monitored at the single cell level and compared between mesophyll protoplasts of HEs and NHEs after Cd treatment. Fluorescence signals denoting H_2_O_2_ were much stronger in protoplasts from NHEs than in those from HEs, at each time point. The generation of H_2_O_2_ was clearly evident as bright green fluorescence. H_2_O_2_ diffused into vacuole compartments in NHE protoplasts after 30 min of Cd exposure, with H_2_O_2_ levels reaching the highest at 1 h (Fig. 8A). In the HE protoplasts, Cd-induced green fluorescence was far less pronounced and was first observed in the plasma membrane and subsequently localized largely to chloroplasts (Fig. 8A). Quantification analysis of H_2_O_2_ production Fig. 8B), measured as relative fluorescence as a percentage of control, showed that H_2_O_2_ levels were slightly, but not significantly, higher in the Cd-treated HE protoplasts compared with controls. In NHE protoplasts H_2_O_2_ formation occurred on a rapid timescale when under Cd stress and was significantly (*P* < 0.01) higher than that of controls at 30 min and 60 min (Fig. 8B). Fluorescence imaging of superoxide failed due to interference from chloroplast autofluorescence.

## Discussion

In hyperaccumulators, the enhanced ability to protect roots from metal toxicity is facilitated partially through efficient shuttling of metals to the shoots (Krämer, 2010). Our previous studies clearly showed a greatly enhanced rate of root-to-shoot translocation was pivotal in the Cd hyperaccumulator *S. alfredii* (Lu *et al.*, 2008). The results in the present study confirm the highly enhanced transport of Cd in shoots of this plant species, as indicated by the marked enrichment of Cd within the vascular bundles of its stems and leaves after Cd exposure (Figs 1, 2, 3A, 3B). The presence of metals within vascular systems is generally associated primarily with a xylem mode of transport and delivery of metals to aerial parts (McNear *et al.*, 2005; Tian *et al.*, 2009). The distribution patterns of Cd in stem tissues showed a larger proportion of Cd localized to the stem vascular bundles of HEs after treatments with 10 μM or 100 μM Cd for 7 d (Fig. 1A, 3A). This was not observed in NHEs (Fig. 1A). Based upon Cd signal intensity, the Cd content of stem vascular tissues of HEs was more than 10-fold higher than that of NHEs. This suggests that a considerable amount of Cd has been transported via the xylem vessels of HE *S. alfredii*, probably as a result of its highly efficient xylem loading of Cd in its roots (Lu *et al.*, 2008; Lu *et al.*, 2009). The efficacy of Cd transport within xylem tissues and into shoots is further confirmed by the enrichment of Cd within the vascular tissues of young stems collected from HE *S. alfredii* plants treated with 100 μM Cd for 30 d (Fig. 2A).

Tissue- and age-dependent variations in Cd distribution patterns in stems of HE *S. alfredii* were also identified in this study, similar to that previously reported for Zn in this plant species (Lu *et al.*, 2014). Vascular-enriched Cd was observed in stems treated with Cd for 7 d and young stems treated with Cd for 30 d, while Cd localization to the pith tissue and cortex layer was observed in the stems of HE *S. alfredii* exposed to Cd for 14 d or 30 d (Fig. 2B, 3B). This is consistent with previously reported results (Tian *et al.*, 2011). Cd signal intensity in stem parenchyma cells of HE *S. alfredii* is more than 10-fold higher than those in vascular tissues of old stems after 30 d of Cd exposure, implying that sequestration of Cd is probably an active process in these tissues. This result is consistent with our previous work, which indicated that Cd is highly accumulated in the pith and cortex of stems and the mesophyll of leaves (Tian *et al.*, 2011). The age- and tissue-dependent variation of Cd-enriched sites in stems implies efficient movement of Cd from vascular bundles into the pith and cortex during its translocation in shoots of HE *S. alfredii*. The parenchyma cells in these tissues, as well as leaf mesophyll (Tian *et al.*, 2011), may serve specifically as terminal storage sites for Cd in this hyperaccumulator plant species. This result is quite different from that reported for the other hyperaccumulators, such as *Noccaea Caerulescens* (formerly *Thlaspi caerulescens*) and *Arabidopsis halleri*, where non-photosynthetic cells of the epidermis or trichomes accumulated the highest levels of Cd (Küpper *et al.*, 2000; Cosio *et al.*, 2005) and there was a higher uptake rate of Cd into epidermal storage cells when compared with mesophyll cells (Leitenmaier and Küpper, 2011). A possible explanation for Cd hyperaccumulation in HE *S. alfredii* is that parenchyma cells in its shoot may have enhanced storage capacity for Cd sequestration.

To evaluate the characteristics of Cd uptake by parenchyma cells in the shoots of HE *S. alfredii*, mesophyll protoplasts were isolated from the leaves of these plants and compared with those from NHE leaves. The results suggested metabolically dependent Cd uptake by mesophyll protoplasts of the two ecotypes, without significant differences in Cd accumulation. Transport of metal ions is generally an active process, which requires an energy supply as a driving force and selective binding sites (Marschner, 1995). Furthermore, metabolically dependent uptake of metals by plants is essentially inhibited at low temperatures (Zhao *et al.*, 2002). The significant inhibition of Cd uptake at low temperatures in both the time- and concentration-dependent kinetics experiments (Figs 4, 5) suggests Cd uptake into the mesophyll protoplasts of both *S. alfredii* ecotypes is metabolically dependent. However, there was little difference in the time- and concentration-dependent accumulation of Cd into mesophyll protoplasts between the two ecotypes (Figs 4, 5). This suggests that the high capacity of Cd sequestration and tolerance in shoots of HE *S. alfredii*, at least for leaves, cannot be solely explained by its rapid cellular uptake rates. Similar results were reported for Cd uptake by leaf protoplasts of the hyperaccumulators *N. caerulescens* and *A. halleri* (Cosio *et al.*, 2004; Ebbs *et al.*, 2009), showing the absence of constitutively high transport capacities for Cd at the level of leaf protoplasts.

A significant aspect of the differences between the two *S. alfredii* ecotypes is Cd transport into the vacuoles of mesophyll protoplasts. In most hyperaccumulators, the metal is sequestered preferentially into compartments where they cannot impair metabolic processes (Küpper and Leitenmaier, 2013). It makes sense for plants to store metals in their vacuoles since this organelle only contains enzymes such as phosphatases, lipases, and proteinases, which have not been identified as targets of heavy metal toxicity (Wink, 1993; Choppala *et al.*, 2014; Zhao *et al.*, 2014). It has been reported in the hyperaccumulator *N. caerulescens* (Ganges ecotype) that Cd accumulated in the cytoplasm a few minutes after its addition and was then transported into vacuoles within its leaf cells (Leitenmaier and Küpper, 2011). This is strongly supported by our observations of Cd distribution patterns in protoplasts of HE *S. alfredii* imaged using Leadmium^TM^ Green AM dye. Cadmium ion transport into vacuoles of protoplasts was not observed in NHEs, while Cd sequestration into vacuoles was consistently observed in protoplasts of HEs after 90–120 min exposure to Cd (Fig. 6, Supplementary Figs S2-S5). This suggests that Cd ions were rapidly transported into vacuoles for storage, providing an efficient form of protection for the functional mesophyll cells in shoots of HE *S. alfredii*. This is supported by the results of viability and membrane integrity experiments (Fig. 7, Supplementary Figs S6-S7), and by H_2_O_2_ imaging (Fig. 8) of Cd-treated mesophyll cells from the two ecotypes, which showed significantly higher Cd tolerance in HE mesophyll protoplasts than those of NHEs.

Here μ -XRF images of Cd in stems of HE *S. alfredii* (Figs. 1–3), together with our previous studies (Tian *et al.*, 2011), indicate that large amounts of Cd are stored in the pith and cortex cells after long-term Cd exposure. The essentially uniform distribution patterns of Cd in the pith and cortex cells of young stems shown by high resolution images (Fig. 3B, Supplementary Fig. S8) further implied that considerable amounts of Cd are efficiently localized to the vacuoles of these parenchyma cells. It should be noted that freeze drying of the plant samples may have shifted dissolved Cd from the vacuole to the nearest available surface, hence localization of Cd in cell walls of the young stems in Fig. 2 is probably an artifact of sample preparation. As a succulent plant, *S. alfredii* has exceptionally large vacuoles in its parenchyma cells, making Cd storage there safer than it would be in regular sized mesophyll cells of other hyperaccumulators (Küpper and Leitenmaier, 2013). It is therefore logical that the efficacy of vacuolar sequestration by the parenchyma cells in shoots of HE *S. alfredii* is an important aspect of metal homeostasis and tolerance in this Cd hyperaccumulator plant species.

Taken together, the results in the present study clearly demonstrate that one of the primary factors responsible for high Cd accumulation in HE *S. alfredii* is highly efficient root-to-shoot translocation, as suggested by the much enhanced Cd signal in the vascular bundles of its young stems. Furthermore, the efficient storage of Cd in vacuoles of the parenchyma cells, as opposed to its rapid transport into protoplasts, may represent a pivotal process in metal homeostasis and tolerance of cells in shoots of the Cd hyperaccumulator HE *S. alfredii*. Combined with the idea that cellular uptake and sequestration of Cd are active processes within the terminal storage sites of HE *S. alfredii*, this study suggests that efficient transport across the tonoplast membranes within the parenchyma cells is the driving force for Cd hyperaccumulation. This provides insights into specific translocation and storage strategies for Cd hyperaccumulation in plants, particularly succulents that have large vacuoles in the thickened and fleshy leaf mesophyll.

## Supplementary Data

Supplementary data are available at *JXB* online.

**Table S1.** Biomass of HE and NHE *S. alfredii* under Cd exposure.

**Fig. S1.** The protoplasts isolated from young leaves of HE *S. alfredii.*

**Fig. S2.** Time-dependent fluorescence imaging of Cd in mesophyll protoplasts isolated from young leaves of HE and NHE of *S. alfredii* using Leadmium^TM^ Green AM dye.

**Fig. S3.** Concentration-dependent fluorescence imaging of Cd in mesophyll protoplasts isolated from young leaves of HE and NHE of *S. alfredii* using Leadmium^TM^ Green AM dye.

**Fig. S4.** Typical images of Cd fluorescence in mesophyll protoplasts isolated from young leaves of HE and NHE of *S. alfredii* treated with 10 µM Cd

**Fig. S5.** Typical images of Cd fluorescence in mesophyll protoplasts isolated from young leaves of HE and NHE of *S. alfredii* after 200 µM Cd exposure for 0–120 min.

**Fig. S6.** Integrities of mesophyll protoplasts (% of control) isolated from HE and NHE *S. alfredii* with 10 μM Cd exposure for 0–120 min.

**Fig. S7.** Viability of mesophyll protoplast isolated from HE and NHE *S. alfredii* after treatments of 200 Cd for 0–120 min as determined using FDA dye.

**Fig. S8.** Nano-XRF imaging of Cd in the cortex tissues of young stem collected from HE *S. alfredii* treated with 100 µM Cd for 30 d. 

## Supplementary Material

supplementary_table_S1_figures_S1_S8
