# Supplementary material for: Uptake, sequestration and tolerance of cadmium at cellular levels in the hyperaccumulator plant species Sedum alfredii
Source: J Exp Bot. 2017 Apr 12;68(9):2387–98. doi: 10.1093/jxb/erx112 (PMC5853795; doi:10.1093/jxb/erx112)
Supplement: supplementary_table_S1_figures_S1_S8 [file erx112_suppl_supplementary_table_s1_figures_s1_s8.pdf]

Supplementary data

Article title: **Uptake, sequestration and tolerance of cadmium at cellular levels in a hyperaccumulator plant species *Sedum alfredii***

Authors: Shengke Tian, Ruohan Xie, Haixin Wang, Yan Hu, Dandi Hou, Xingcheng Liao, Patrick H. Brown, Hongxia Yang, John M. Labavitch, and Lingli Lu*

Table S1 Biomass of HE and NHE *S. alfredii* exposed to different Cd levels for 7-30 d

Cd levels (μM)		Biomass (mg $plant^{-1}$)					
		Roots			Shoots		
		**7 d**	**14 d**	**30 d**	**7 d**	**14 d**	**30 d**
**HE**	**0**	40.2 ±0.9a	46.9 ±4.9a	67.5 ±6.0a	268.9 ±19.4a	396.1 ±26.7a	748.4 ±76.6a
	**10**	41.9 ±1.7a	48.3 ±3.6a	76.5 ±6.8a	253.4 ±26.1a	401.3 ±41.3a	750.6 ±91.4a
	**100**	38.9 ±2.5a	45.8 ±4.8a	60.1 ±5.2a	263.6 ±55.6a	388.9 ±37.6a	685.0 ±61.7a
**NHE**	**0**	42.2 ±5.0a	50.6 ±7.1a	77.7 ±10.3a	238.0 ±23.1a	365.1 ±39.0a	725.5 ±79.0a
	**10**	36.3 ±3.8b	43.2 ±6.8a	56.1 ±8.2b	203.0 ±11.0b	267.8 ±42.0b	343.6 ±23.6b
	**100**	34.1 ±3.5b	-	-	137.3 ±18.3c	-	-

All data represent the means ± SE of 4 replicates. Different letters indicate significance of the treatments at $P < 0.05$ for the same ecotype. Plants of NHE didn't grow at 100 μM after 14 d exposure.

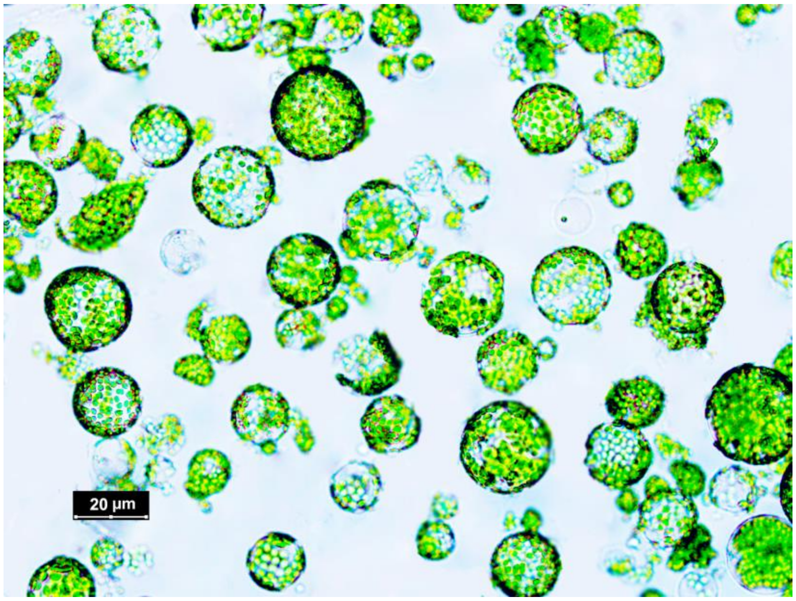

Fig. S1 The protoplasts isolated from young leaves of HE *S. alfredii*.

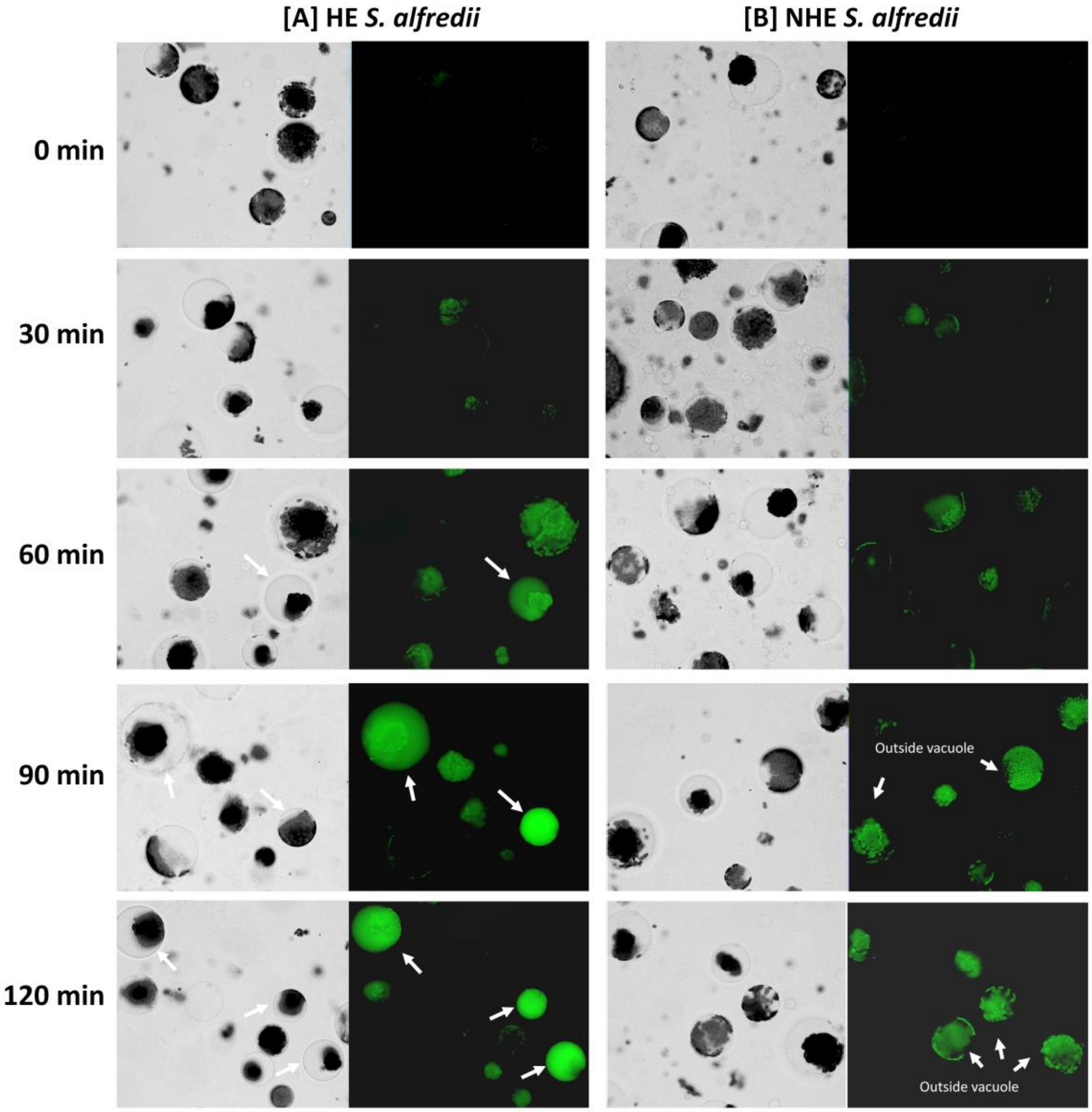

Fig. S2 Time-dependent fluorescence imaging of Cd in mesophyll protoplasts isolated from young leaves of HE (A) and NHE (B) of *S. alfredii* by using Leadmium™ Green AM dye. The protoplasts were isolated from young leaves of 4-week old HE and NHE *S. alfredii*, and pre-loaded with Leadmium™ Green AM dye for 30 min, were treated with 10 µM Cd for 0, 10, 30, 60, 90, and 120 min. Green fluorescence in images represents the binding of the dye to Cd. Scale bar: 20 µm.

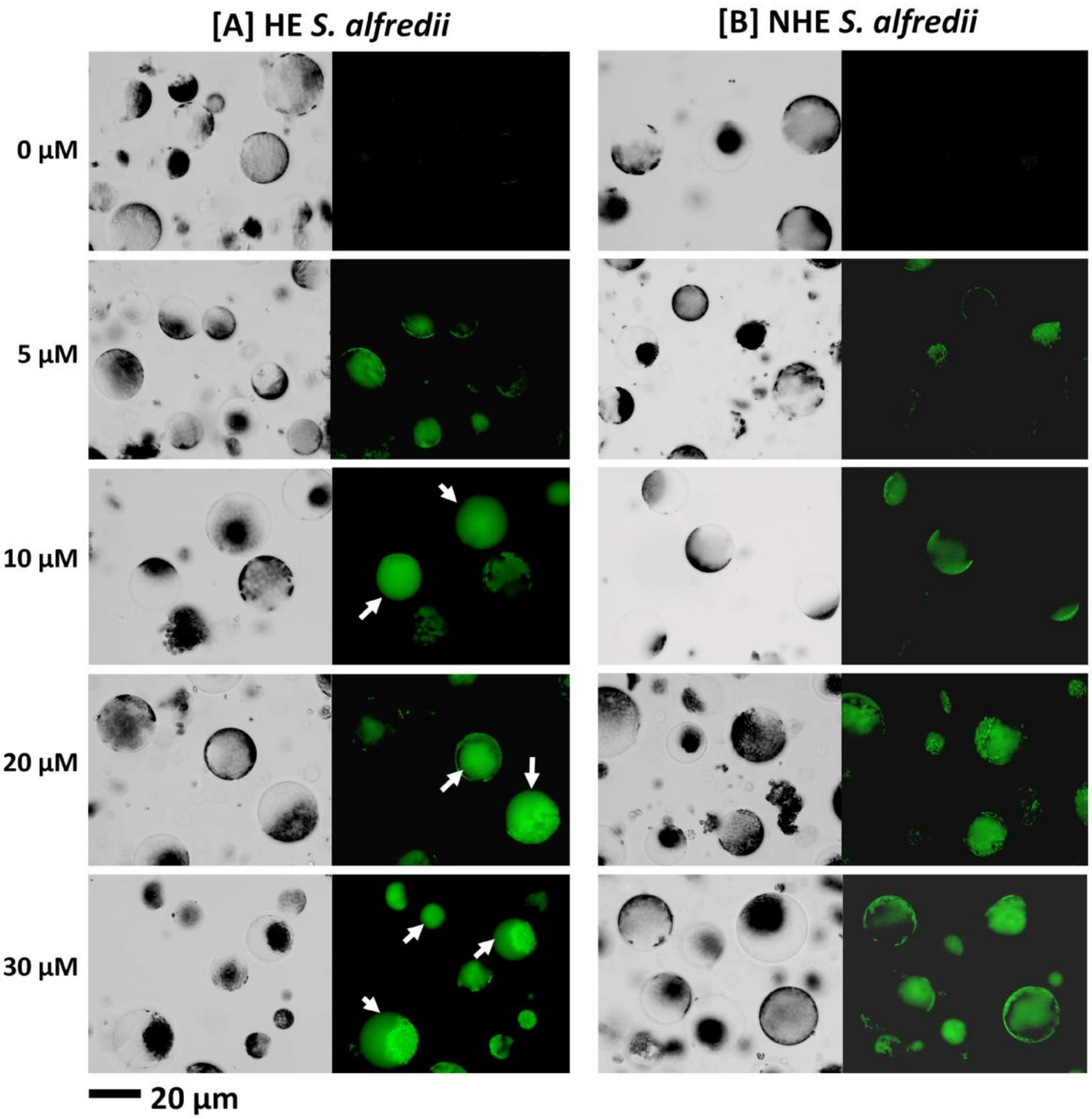

Fig. S3 Concentration-dependent fluorescence imaging of Cd in mesophyll protoplasts isolated from young leaves of HE (A) and NHE (B) of *S. alfredii* by using Leadmium™ Green AM dye. The protoplasts were isolated from young leaves of 4-week old HE and NHE *S. alfredii*, and pre-loaded with Leadmium™ Green AM dye for 30 min, were treated with 0, 5, 10, 20, 30 μM Cd for 90 min. Green fluorescence in images represents the binding of the dye to Cd.

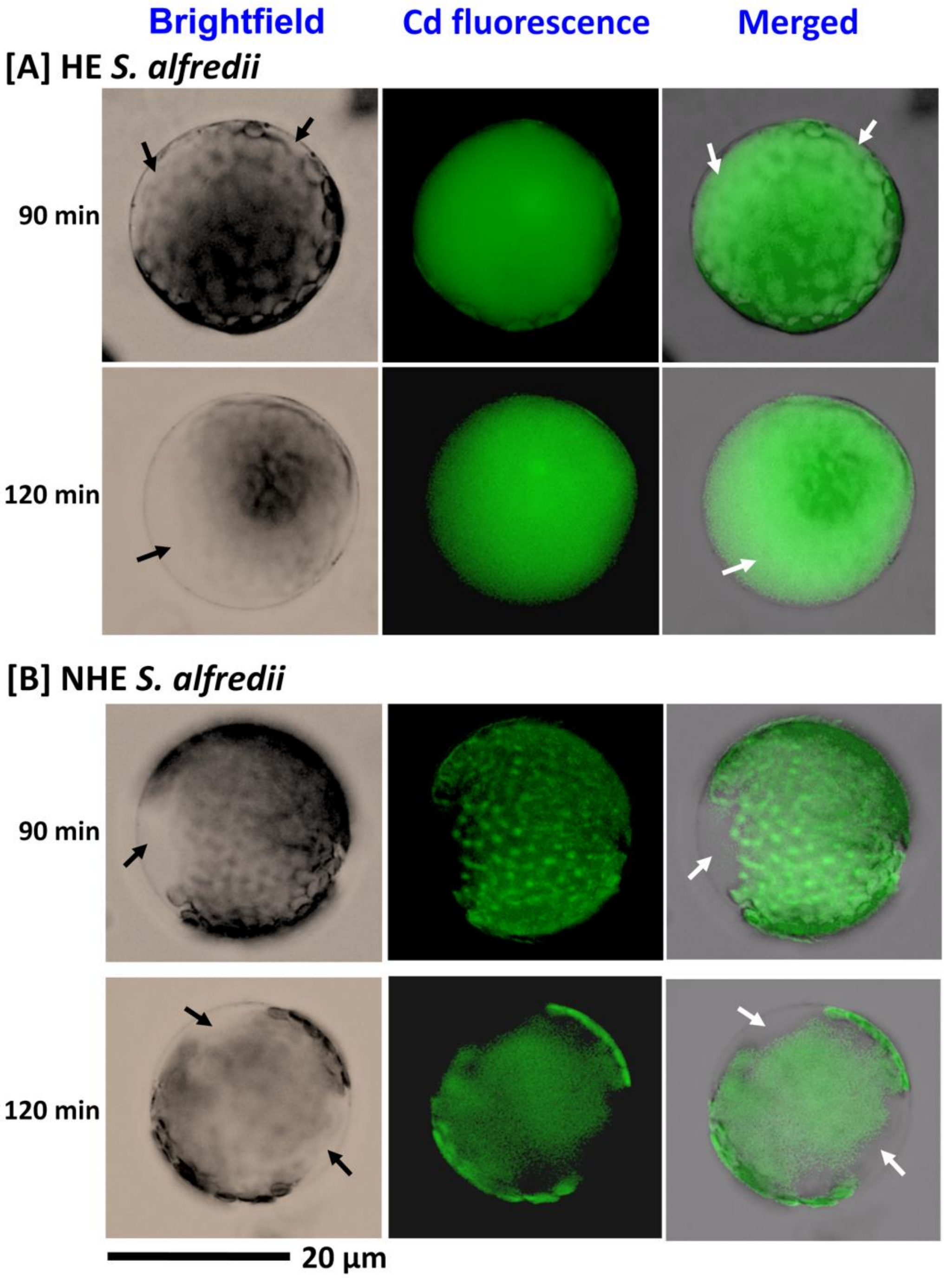

Fig. S4 Typical images of Cd fluorescence in mesophyll protoplasts isolated from young leaves of HE (A) and NHE (B) of *S. alfredii* treated with 10 µM Cd. The protoplasts were isolated from young leaves of 4-week old HE and NHE *S. alfredii*, and pre-loaded with Leadmium^{TM} Green AM dye for 30 min, were treated with 10 µM Cd for 90 min, and 120 min. Green fluorescence in images represents the binding of the dye to Cd. Scale bar: 20 µm.

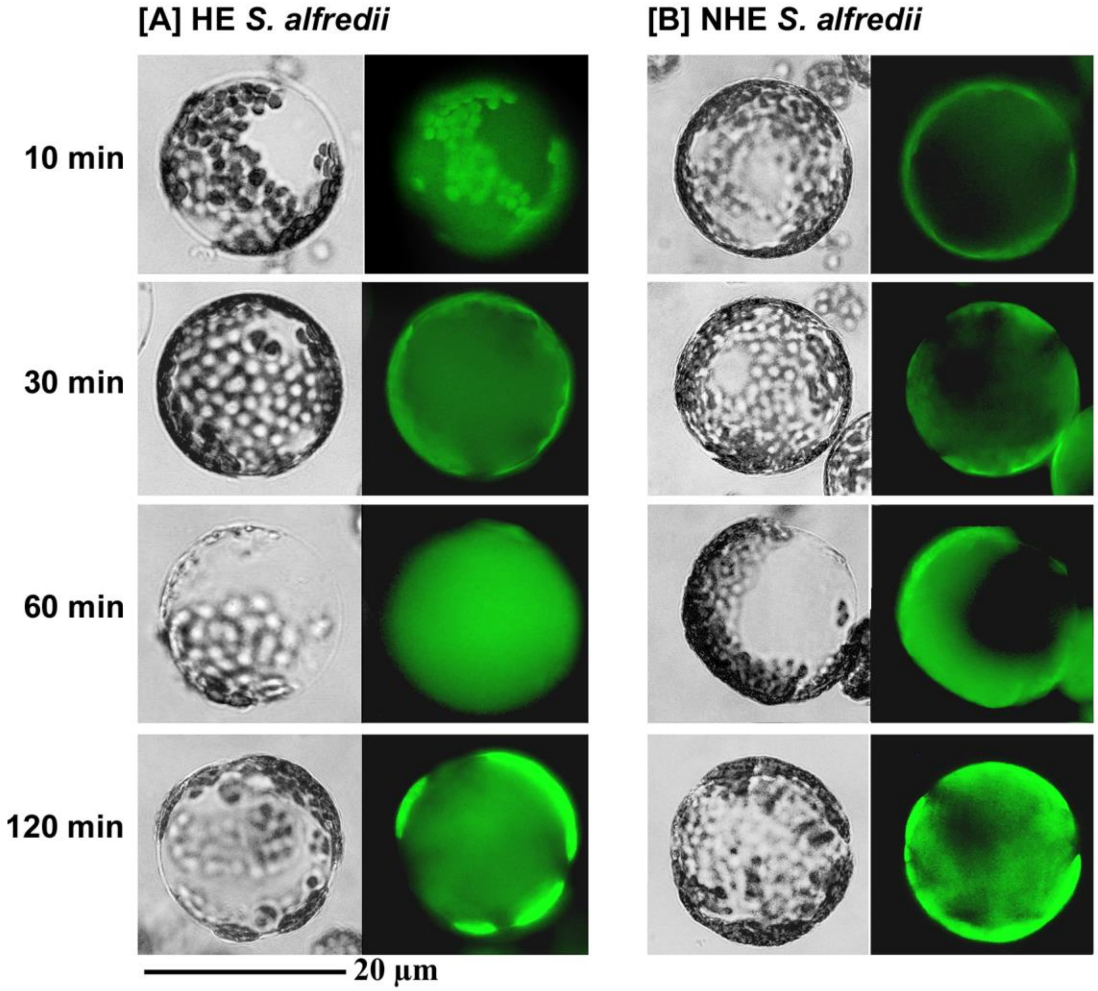

Fig.

Fig. S5 Typical images of Cd fluorescence in mesophyll protoplasts isolated from young leaves of HE (A) and NHE (B) of *S. alfredii* after 200 µM Cd exposure for 0-120 min. The protoplasts were isolated from young leaves of 4-week old HE and NHE *S. alfredii*, and pre-loaded with Leadmium^{TM} Green AM dye for 30 min, were treated with 200 µM Cd for 0 min, 10 min, 30 min, 1.0 h, and 2.0 h. Green fluorescence in images represents the binding of the dye to Cd. Scale bar: 20 µm.

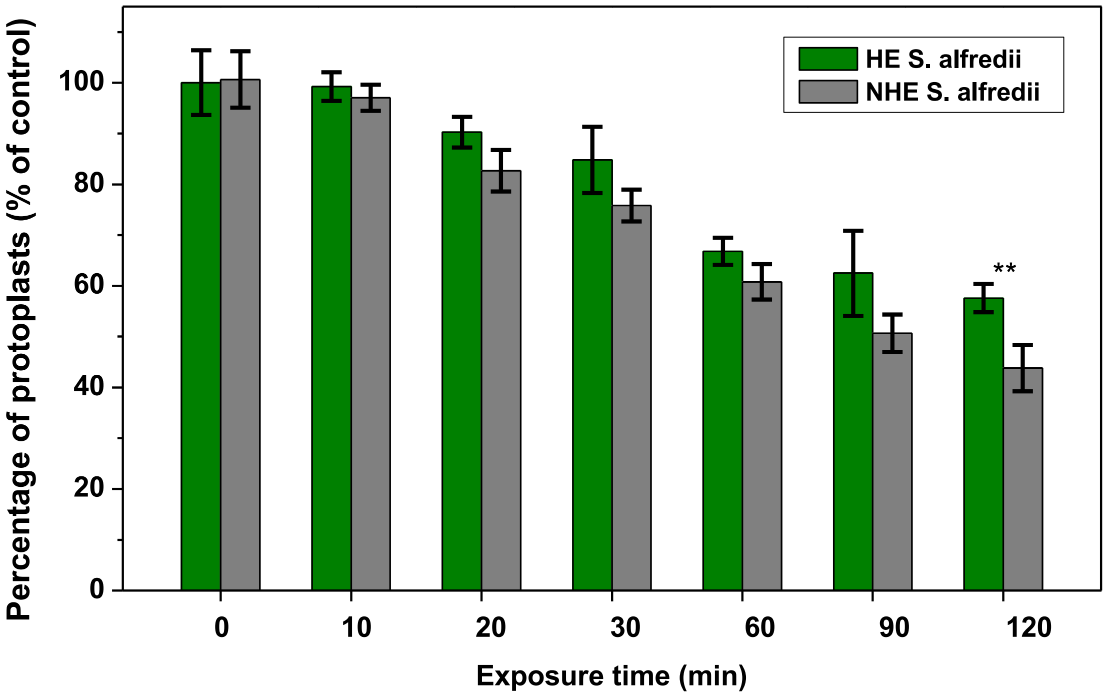

Fig. S6 Integrities of mesophyll protoplasts (% of control) isolated from HE and NHE *S. alfredii* with 10 μM Cd exposure for 0-120 min. The protoplasts were isolated from young leaves of 4-week old HE and NHE *S. alfredii*, and treated with 10 μM Cd in solutions for different periods as shown in the figure. Data points and error bars represent means (n=5) and SE, respectively. One and two asterisks indicate significant difference between HE and NHE treatments at $P < 0.05$ and $P < 0.01$, respectively.

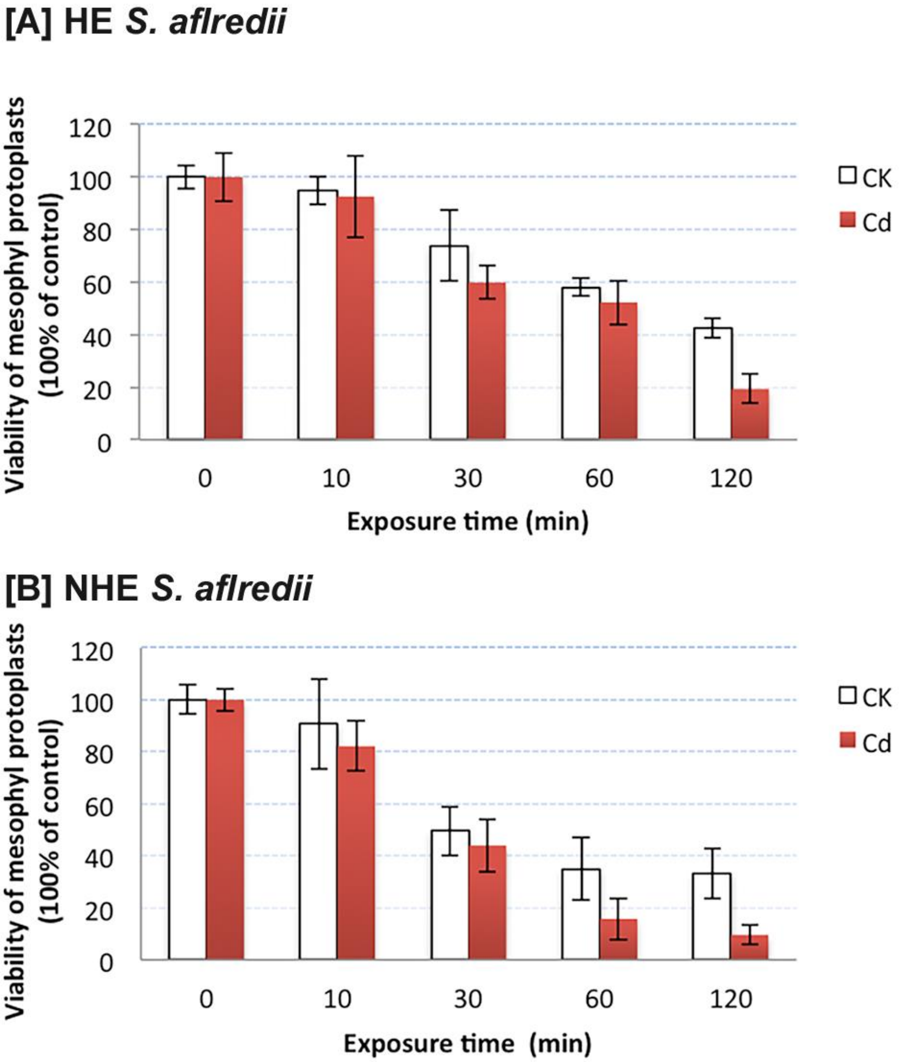

Fig. S7 Viability of mesophyll protoplast isolated from HE (a) and NHE (b) *S. alfredii* after treatments of 200 μM Cd for 0-120 min as determined by using FDA dye. The protoplasts were isolated from young leaves of 4-week old HE and NHE *S. alfredii*, and treated with 200 μM Cd in solutions for different periods as shown in the figure. Data points and error bars represent means (n=10) and SE, respectively.

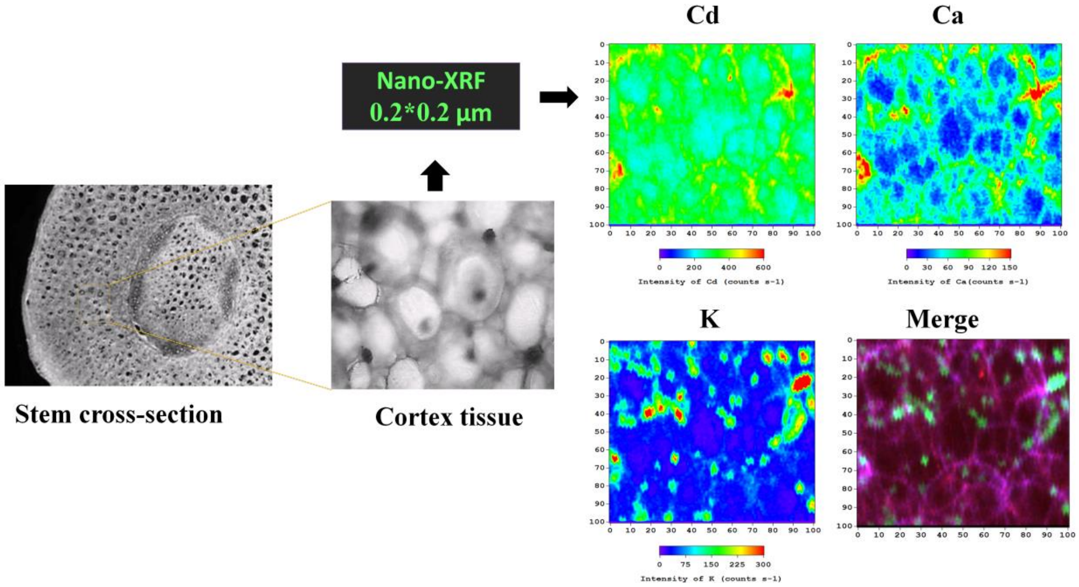

Fig. S8 Nano-XRF imaging of Cd in the cortex tissues of young stem collected from HE *S. alfredii* treated with 100 μM Cd for 30 D. The microscope images show the stem region selected for μ-XRF imaging. The red color, depicting elemental concentrations in each map, was scaled to the maximum value for each map.

High resolution Synchrotron measurements

High-resolution x-ray fluorescence was performed at a helium atmosphere on the Advanced Photon Source 2-ID-D hard X-ray microprobe beamline (Cai *et al.,* 2003). Incident x-rays of 28 keV were chosen to excite elements from K to Cd. A Fresnel zone plate focused the x-ray beam to a spot size of 0.2×0.2 μm on the sample, which was raster scanned at resolutions of 1 μm step in the sample image, with dwell times ranging from 0.5s per pixel. X-ray fluorescence from the sample was captured with an energy-dispersive silicon drift detector. The resulting elemental maps were visualized and analyzed with the program MAPS (Vogt, 2003).

Reference

Cai Z, Lai B, Xiao Y, Xu S. 2003. An X-ray diffraction microscope at the Advanced Photon Source. *Journal De Physique Iv* **104**: 17-20.

Vogt S. 2003. MAPS: A set of software tools for analysis and visualization of 3D X-ray fluorescence data sets. *Journal De Physique Iv* **104**: 635-638.
